# Herbicide-tolerant endophytic bacteria of rice plants as the biopriming agents for fertility recovery and disease suppression of unhealthy rice seeds

**DOI:** 10.1186/s12870-019-2206-z

**Published:** 2019-12-23

**Authors:** Chakrapong Rangjaroen, Saisamorn Lumyong, William T. Sloan, Rungroch Sungthong

**Affiliations:** 1 Department of Agricultural Management Technology, Faculty of Science and Technology, Phranakhon Rajabhat University, Bangkok, 10220 Thailand; 2 Microbiology Division, Department of Biology, Faculty of Science, Chiang Mai University, Chiang Mai, 50200 Thailand; 3 Center of Excellence in Microbial Diversity and Sustainable Utilization, Chiang Mai University, Chiang Mai, 50200 Thailand; 4 Academy of Science, The Royal Society of Thailand, Bangkok, 10300 Thailand; 5 Infrastructure and Environment Research Division, School of Engineering, University of Glasgow, Glasgow, G12 8LT UK

**Keywords:** Bacterial endophytes, Phytopathogenic fungi, Dirty panicle disease, Rice, Seed stock, Seed biopriming, Herbicide-tolerant bacteria

## Abstract

**Background:**

Dirty panicle disease (DPD) caused by several fungal phytopathogens results in damage and depreciation of rice seeds. Unhealthy rice seeds with DPD are potent reservoirs of pathogens and unable to be used as seed stock as they can spread the disease in the paddy fields leading to the severe loss of rice yield and quality. In this study, we aim to search for beneficial endophytes of commercially cultivated rice plants and utilize them as biostimulants in seed biopriming for fertility recovery and disease suppression of unhealthy rice seeds.

**Results:**

Forty-three bacterial endophytes were isolated from rice plants grown in the herbicide-treated paddy fields. Five isolates of these endophytes belonging to the genus *Bacillus* show excellent antifungal activity against fungal pathogens of DPD. Based on germination tests, biopriming unhealthy rice seeds by soaking in bacterial suspensions for 9 or 12 h was optimal as evidenced by the lowest disease incidence and longer shoot and root lengths of seedlings germinated, compared with controls made of non-treated or hydroprimed healthy and unhealthy seeds. Pot experiments were carried out to evaluate the impact of seed biopriming, in which the percentage of healthy rice yield produced by rice plants emerging from bioprimed seeds was not significantly different, compared to the controls originating respectively from non-treated healthy seeds and chemical fungicide-treated unhealthy seeds.

**Conclusion:**

Biopriming of unhealthy rice seeds with herbicide-tolerant endophytic bacteria could recover seed fertility and protect the full life cycle of emerging rice plants from fungal pests. With our findings, seed biopriming is a straightforward approach that farmers can apply to recover unhealthy rice seed stock, which enables them to reduce the cost and use of agrochemicals in the commercial production of rice and to promote green technology in sustainable agriculture.

## Background

Thai jasmine rice (*Oryza sativa* L. cv. KDML105) is a commercially important crop in Thailand, and its recent export value rises to 1225$ t^− 1^ [1]. The quality of rice grains is the critical factor that determines the commercial value of this cereal product. Some of the high-quality, healthy-looking rice seeds are kept and used as seed stock for subsequent cultivation. Rice seeds that are unhealthy by having abnormal changes and or lesions are sorted out, and depending upon their quality, these unhealthy seeds may be used as animal feed or left for destruction. The physical appearances of unhealthy rice seeds are the symptoms of dirty panicle disease (DPD) or grain discoloration, which is a typical disease of rice seeds caused by several phytopathogenic fungi [2]. Hence, unhealthy rice seeds are considered the potent reservoirs of phytopathogens and unable to be used as seed stock because they can spread the disease in the paddy field leading to the severe loss of rice yield and quality.

It is hard to detect and prevent DPD earlier, as the causative agents are nonspecific, and the physical symptoms occur on mature rice grains at the harvesting phase. When DPD occur during rice production, it is often too late to recover the quality of rice yields. Improper storage of healthy-looking rice seeds can also induce DPD because the surface of rice seeds house more than 100 species of fungi and numerous of them are common pathogens of rice [3, 4]. In 2013, massive rice yields produced in Thailand became invaded with fungal pests during storage [5], while a lot of these rice seeds had symptoms of DPD. This damage did not only engender an adverse impact on the country’s agro-economy but also the profits of farmers. A strategy to prevent the occurrence of DPD is to use good quality seed stock for cultivation of rice. In order to maintain the quality of seed stock before use in rice cultivation, seed treatment is often performed.

Although many approaches are available in the market for seed treatment [6, 7], most are carried out for maintaining healthy seeds and protecting them from pest invasion during storage. Unfortunately, when a little sign of DPD observed on rice seeds, they have been treated as unhealthy seeds and become agricultural waste. Recovery of these unhealthy rice seeds for being used as seed stock with acceptable quality would be a challenging way to add value to them and further reduce the cost in rice cultivation. Hitherto, the optimal treatment for fertility recovery and disease suppression of unhealthy rice seeds is still unknown. Chemical treatment of these unhealthy seeds with pesticides may be an approach for rapid disease suppression. However, using pesticide-treated seeds for cultivation may result in some environmental concerns, as the residues of pesticides may endanger the beneficial plant-associated microbes and persist in agroecosystem or accumulate into the ecological food web [6, 8]. Moreover, pesticide-contaminated crops can depreciate their quality and adversely affect consumers’ health [9, 10].

Seed biopriming could be a strategy of green technologies for seed treatment in promoting sustainable agriculture with the aim of reducing the use of agrochemicals in crop production [11–13]. This biological technique employs beneficial microbes possessing plant growth-promoting (PGP) potentials, by simply saturating seeds into an active microbial suspension for an optimal duration followed by seed moisture monitoring before use as seed stock. The method allows microbial cells to colonize the outer parts of seeds, where the synergistic interactions take place as microbes utilize grains’ nutritive exudates for their proliferation and biosynthesis of biocontrol agents to protect seeds from pests [13, 14]. Numerous PGP bacteria have been used as bioactive agents for biopriming [11–14]. These bacteria are often isolated from plant-associated sources, like PGP rhizobacteria that live in the plant rhizosphere. Another technical advantage of using PGP bacteria in biopriming is that they are fast growing and easy for harvesting and preparing as microbial suspension. By the way, the efficiency of seed biopriming for the restoration of unhealthy rice seeds is yet to be investigated.

It is conceivable that many steps in rice production require beneficial plant-microbe interactions to promote growth and productivity of rice plants. Many studies revealed that several rice endophytic bacteria are promising growth promoters of rice plant [15–18]. These endophytes live in the interiors of rice and do not form any disease. Moreover, they can either promote rice growth or protect rice from invasive phytopathogens for the entire period of rice development and production. However, in commercial rice cultivation, it is hard to avoid using agrochemicals in order to maintain high rice yield [16, 19]. For instance, in the paddy field, weeds are unwanted as they grow in competition with rice plants, by stealing soil nutrients and water [20]. Weeds can also be reservoirs of pests and phytopathogens that damage rice plants and yields [21]. Hence, herbicides are often applied in the paddy field to stop growth of weeds. With these reasons, endophytic PGP bacteria with capability to tolerate herbicides commonly used in rice production would potentially be the ideal biopriming agents.

In this study, we aim to develop a straightforward protocol for biopriming of unhealthy rice seeds using endophytic PGP bacteria of rice plants. The virulence level of DPD of stored rice seed stock was classified. Rice seeds with severe DPD served as the sources for isolation of fungal pathogens. Rice plants grown commercially in fields treated with different herbicides were used for isolation of herbicide-tolerant endophytic bacteria. Antifungal activity of the isolated endophytic bacteria was screened. Biopriming of unhealthy rice seeds using the best antifungal bacteria was developed and assessed for its effectiveness in fertility recovery and disease suppression of the rice seeds. The assessments were carried out from germination to harvesting of rice yields.

## Results

### Fungal pathogens of DPD

**Fig. 1:**
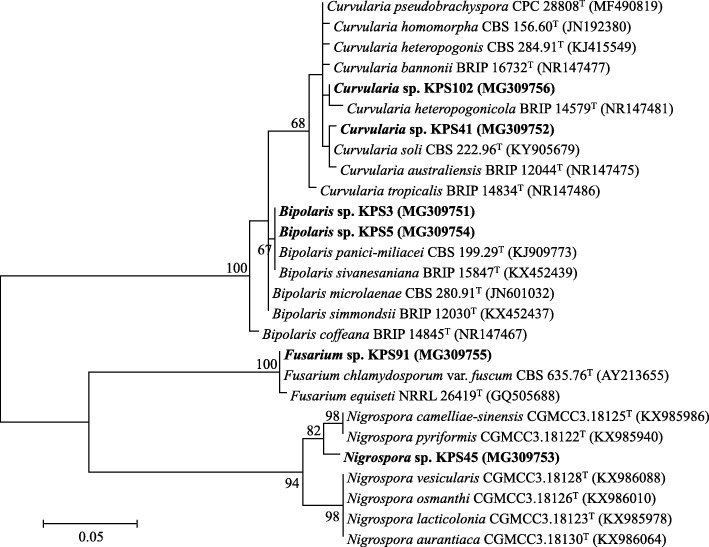
Unrooted phylogenetic tree of ITS gene sequences of the virulent pathogenic fungi isolated from rice seeds showing dirty panicle disease. The fungal isolates are in bold, and their GenBank accession numbers are in parentheses. The tree was constructed using the Maximum-Likelihood algorithm. Bootstrap values (based on 1000 replications) of > 60% are at the tree’s nodes, and scale bar represents 5% dissimilarity

Sixty-two fungi were isolated from rice seeds showing DPD, six of which exhibited virulent pathogenicity (Table 1). These pathogenic fungi were identified as the members of the genera *Bipolaris*, *Curvularia*, *Nigrospora*, and *Fusarium*, supported by 97–100% similarity of their ITS gene sequences compared to those available in public databases (Table 1 and Fig. 1). Two isolates (KPS3 and KPS5) belonged to the genus *Bipolaris*, and both were closely related to *Bipolaris panici-miliacei* CBS 199.29^T^ supported by 100% similarity of the gene sequences. The other two isolates, KPS41 and KPS102, belonged to the genus *Curvularia*, and each of them was closely related to *Curvularia soli* CBS 222.96^T^ and *Curvularia pseudobrachyspora* CPC28808^T^ supported by 99 and 97% similarity of the gene sequences, respectively.

**Table 1:** Pathogenic fungi of Thai jasmine rice seeds^*a*^ showing dirty panicle disease

Pathogenic fungi^*b*^	Closest phylogenetic species^*c*^	% Identity^*c*^
KPS3 (MG309751)	*Bipolaris panici-miliacei* CBS 199.29^T^ (KJ909773)	100
KPS5 (MG309754)	*B*. *panici-miliacei* CBS 199.29^T^ (KJ909773)	100
KPS41 (MG309752)	*Curvularia soli* CBS 222.96^T^ (KY905679)	99
KPS45 (MG309753)	*Nigrospora camelliae*-*sinensis* CGMCC3.18125^T^ (KX985986)	98
KPS91 (MG309755)	*Fusarium chlamydosporum* var. *fuscum* CBS 635.76^T^ (AY213655)	99
KPS102 (MG309756)	*Curvularia pseudobrachyspora* CPC28808^T^ (MF490819)	97

^*a*^The plant specimen was deposited for further reference at CMUB Herbarium with the code, CMUB39907. ^*b*^The fungal ITS gene sequences were deposited in GenBank with accession numbers in parentheses. ^*c*^The % identity refers to the similarity percentage of the gene sequence of each fungal isolate compared to its closest phylogenetic species (GenBank accession number)

### Herbicide-tolerant endophytic bacteria of rice and their antifungal activity

**Fig. 2:**
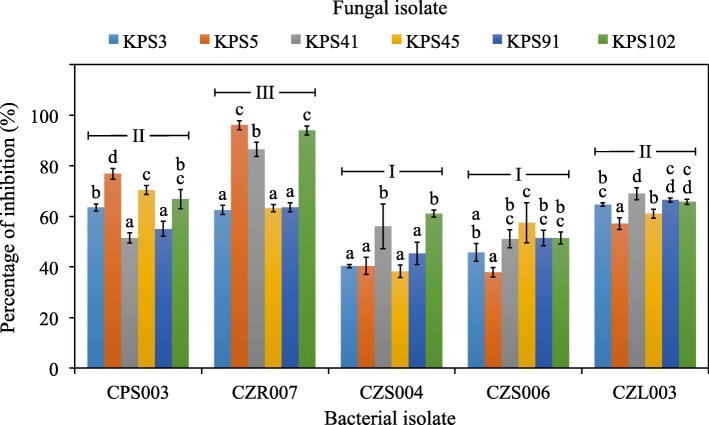
Antifungal activity of herbicide-tolerant endophytic bacteria of field-growing rice. These bacterial isolates (CPS003, CZR007, CZS004, CZS006, and CZL003) showed the best antifungal activity against the virulent pathogenic fungi of rice seeds showing dirty panicle disease (KPS3, KPS5, KPS41, KPS45, KPS91, and KPS102). The percentages of inhibition were calculated using the data derived from the dual culture assay of the test bacteria and fungi. Error bars are mean values ± SDs of four replicates per assay. The statistical differences at *P* = 0.05 of means are indicated with small case letters for each test bacterium against different test fungi and with the Roman numbers for the comparison between different bacterial isolates regardless of the distinct fungi tested. All statistical analyses were conducted using one-way ANOVA with Tukey’s post hoc test

**Fig. 3:**
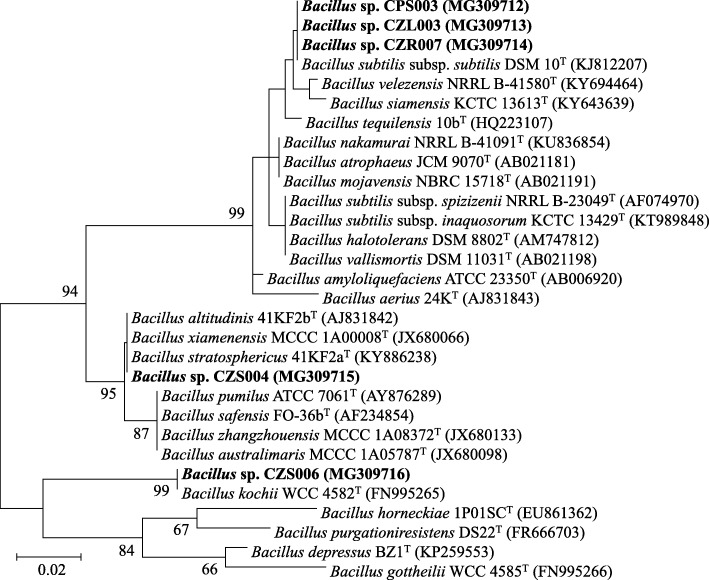
Unrooted phylogenetic tree of 16S rRNA gene sequences of the best antifungal isolates of herbicide-tolerant endophytic bacteria of field-growing rice. The isolated bacteria are in bold, and their GenBank accession numbers are in parentheses. The tree was constructed using the Maximum-Likelihood algorithm. Bootstrap values (based on 1000 replications) of > 60% are at the tree’s nodes, and scale bar represents 2% dissimilarity

**Fig. 4:**
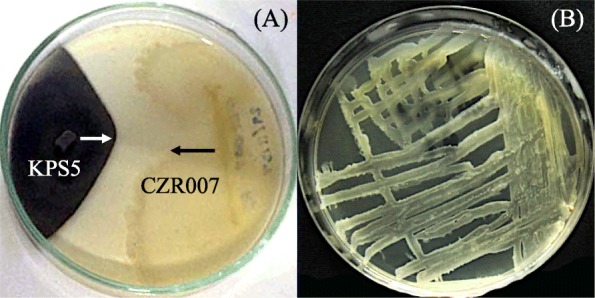
Antifungal activity and herbicide tolerance of *Bacillus* sp. CZR007. The antifungal activity of isolate CZR007 was tested against the fungus, *Bipolaris* sp. KPS5 on potato dextrose agar (**a**). The herbicide tolerance of isolate CZR007 was confirmed by checking viability of the bacterium on Luria-Bertani agar medium supplemented with 1.60 mL of a herbicide, Clomazone (**b**)

Forty-three herbicide-tolerant bacteria were isolated from the interiors of different rice cultivars growing in the herbicide-treated paddy fields (Table 2). *O. sativa* L. var. *indica* cv. RD41 housed the highest number of bacteria (13 isolates), while the other sources, *O. sativa* L. var. *indica* cv. Pathumthani 1 grown at different field locations and treated with different herbicides, each resulted in 10 bacterial isolates. Root tissues of any rice cultivars housed a relatively higher number of bacteria (4–7 isolates). All bacteria were tested for their antifungal activity against a set of virulent fungi causing DPD of rice seeds, and five of which showed excellent antifungal activity (Fig. 2). These bacteria were identified as the members of the genus *Bacillus* supported by 99–100% similarity of their 16S rRNA gene sequences compared to those available in public databases (Table 3 and Fig. 3). Three isolates (CPS003, CZR007, and CZL003) were closely related to *Bacillus subtilis* subsp. *subtilis* DSM 10^T^ supported by 99% similarity of the gene sequences. Each of the other two isolates (CZS004 and CZS006) was closely related to *Bacillus altiludinis* 41KF2b^T^ and *Bacillus kochii* WCC4582^T^ supported by 100 and 99% similarity of the gene sequences, respectively. *Bacillus* sp. CZR007 revealed the highest antifungal activity (nearly 100%) against *Bipolaris* sp. KPS5 and *Curvularia* sp. KPS102 (Figs. 2 and 4).

**Table 2:** Source information of herbicide-tolerant endophytic bacteria of field-growing rice

Rice cultivar	Field location	Herbicide concentration		Plant age (day)^*b*^	Herbicide-tolerant endophytic bacteria (number of isolate)		
		Field application (active ingredient)^*a*^	Isolation medium (L^− 1^)		Root	Stem	Leaf
*Oryza sativa* L. var. *indica* cv. Pathumthani 1	Sam Chuk District, Suphan Buri Province, Thailand	3 g of 10% (w/v) Metsulfuron-methyl + 10% (w/v) Chlorimuron-ethyl in 60 L of water	5 mg Metsulfuron-methyl and 5 mg Chlorimuron-ethyl	13^c^	MCR001 – MCR004 (4)	MCS005 – MCS007 (3)	MCL008 – MCL010 (3)
		40 mL of 10% (w/v) Bispyribac-sodium in 60 L of water	0.07 mL Bispyribac-sodium	16^d^	BSR001 – BSR005 (5)	BSS006 (1)	BSL007 – BSL010 (4)
	Si Prachan District, Suphan Buri Province, Thailand	100 mL of 12% (w/v) Clomazone + 27% (w/v) Propanil in 20 L of water	0.64 mL Clomazone and 1.44 mL Propanil	15^e^	CPR005 – CPR007 (3)	CPS001 – CPS004 (4)	CPL008 – CPL010 (3)
*Oryza sativa* L. var. *indica* cv. RD41	Sai Noi District, Nonthaburi Province, Thailand	200 mL of 48% (w/v) Clomazone in 60 L of water	1.60 mL Clomazone	13^f^	CZR007 – CZR013 (7)	CZS004 – CZS006 (3)	CZL001 – CZL003 (3)

^*a*^The herbicides were applied by spraying onto the field after sowing for 13–16 days and the rice plants were collected from the field 3 days afterward. ^*b*^Plant specimens were deposited for further references at CMUB Herbarium with the following codes, ^*c*^CMUB39903, ^*d*^CMUB39904, ^*e*^CMUB39905, and ^*f*^CMUB39906

**Table 3:** Herbicide-tolerant endophytic bacteria of field-growing rice

Herbicide-resistant endophytic bacteria^*a*^	Closest phylogenetic species^*b*^	% Identity^*b*^
CPS003 (MG309712)	*Bacillus subtilis* subsp. *subtilis* DSM 10^T^ (KJ812207)	99
CZR007 (MG309714)	*B. subtilis* subsp. *subtilis* DSM 10^T^ (KJ812207)	99
CZS004 (MG309715)	*Bacillus altitudinis* 41KF2b^T^ (AJ831842)	100
CZS006 (MG309716)	*Bacillus kochii* WCC4582^T^ (FN995265)	99
CZL003 (MG309713)	*B*. *subtilis* subsp. *subtilis* DSM 10^T^ (KJ812207)	99

^*a*^These herbicide-resistant endophytic bacteria showed the best antifungal activity against pathogenic fungi of dirty panicle disease, and their16S rRNA gene sequences were deposited in GenBank with accession numbers in parentheses. ^*b*^The % identity refers to the similarity percentage of the gene sequence of each bacterial isolate compared to its closest phylogenetic species (GenBank accession number)

### Fertility and quality of unhealthy rice seed stocks after biopriming

**Fig. 5:**
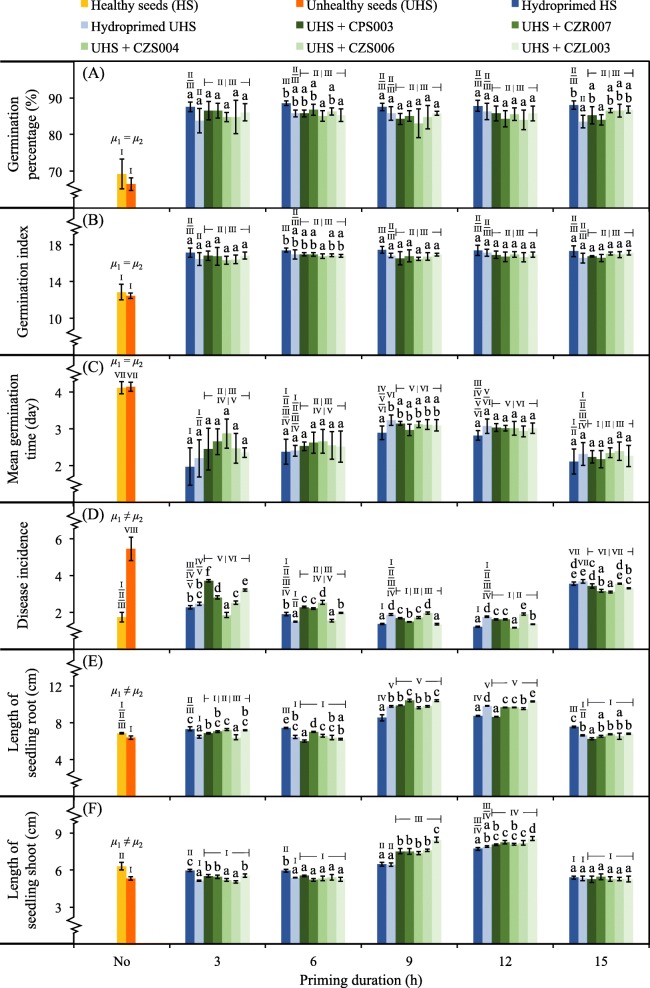
Impacts of seed biopriming on seed germination and seedling growth. Germination percentage (**a**), germination index (**b**), mean germination time (**c**), disease incidence (**d**), and length of seedlings’ roots (**e**) and shoots (**f**) are the evaluating factors. Five bacterial isolates (CPS003, CZR007, CZS004, CZS006, and CZL003) were used for seed biopriming. Bioprimed unhealthy seeds (UHS) were tested in comparison with the controls made of non-treated (No) or hydroprimed healthy (HS) and UHS. Error bars are mean values ± SDs of 100 seeds per each test and with four replicates in total. The statistical comparison of means between HS (*μ*1) and UHS (*μ*2) without priming was conducted using the independent-samples *t*-test at *P* = 0.05. The statistical differences of means compared by one-way ANOVA with Tukey’s post hoc test at *P* ≤ 0.05 are indicated with small case letters for every type of primed seeds (within the same priming duration) and with the Roman numbers for the comparison between any tests and controls. The data derived from any bioprimed seeds were calculated as a mean, regardless of the different bacteria tested

The biopriming potentials of unhealthy rice seeds with selected bacteria were primarily assessed at the germination and seedling growth phases using germination percentage (GP), germination index (GI), mean germination time (MGT), disease incidence (DI), and the length of seedlings’ roots and shoots as the indicators (Fig. 5). GPs of non-treated healthy and unhealthy seeds were not statistically different and showed significantly lower (~ 65–70%) compared to those of any primed seeds (~ 85–90%) (Fig. 5-a). There was no significant difference between GPs of any primed seeds. However, unhealthy seeds hydropriming for 3 and 15 h showed slightly lower GPs. GI also exhibited a similar trend of results, while any seeds priming for 3 h revealed a slightly lower GI (Fig. 5-b). MGTs of non-treated healthy and unhealthy seeds were slowest (~ 4 days), while any seeds priming for 3 and 15 h exhibited slightly faster (Fig. 5-c). DI of non-treated unhealthy seeds was highest (Fig. 5-d). Among any primed seeds, DI was relatively lower when priming for 6, 9, and 12 h. The lengths of seedlings’ roots (Fig. 5-e) and shoots (Fig. 5-f) were longest when such seedlings emerging from unhealthy seeds biopriming for 9 and 12 h. These results suggested that the optimal priming duration (OPD) for biopriming of unhealthy rice seeds was 9 or 12 h.

**Fig. 6:**
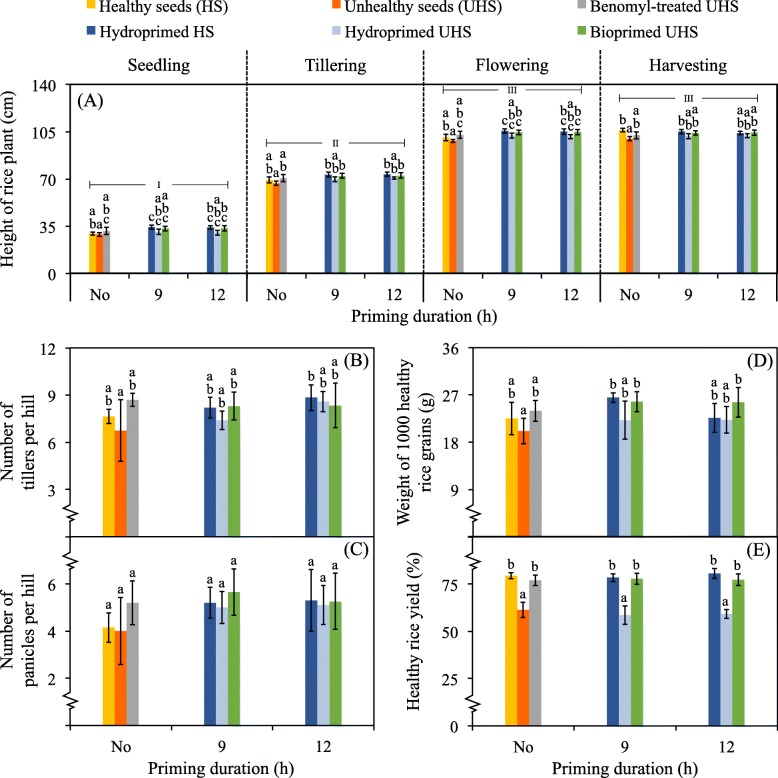
Impacts of seed biopriming on plant growth and development in pot experiments. The experiments were conducted for 4 months, dividing into seedling (day 0–14), tillering (day 30–60), flowering (day 75–100), and harvesting (day 120) phases of rice cultivation. The height of rice plants (**a**) was measured at every phase. At the harvesting stage, the numbers of tillers (**b**) and panicles (**c**) per hill, the weight of 1000 healthy rice grains (**d**), and the percentage of healthy rice yield (**e**) were quantified. The tests are the rice plants arising from unhealthy seeds (UHS) biopriming with any selected bacteria, and those emerging from non-treated (No) or hydroprimed healthy seeds (HS) and UHS plus benomyl-treated UHS are controls. There was no significant difference across the results from different bacterial isolates tested. We, therefore, calculated the global mean value of all data derived from bioprimed tests as one. Error bars are mean values ± SDs of at least 20 replicated measurements. The statistical differences of means compared by one-way ANOVA with Tukey’s post hoc test at *P* ≤ 0.05 are indicated with small case letters for any tests and controls (within the same priming duration), and with the Roman numbers for the comparison of means at different phases in (**a**)

**Fig. 7:**
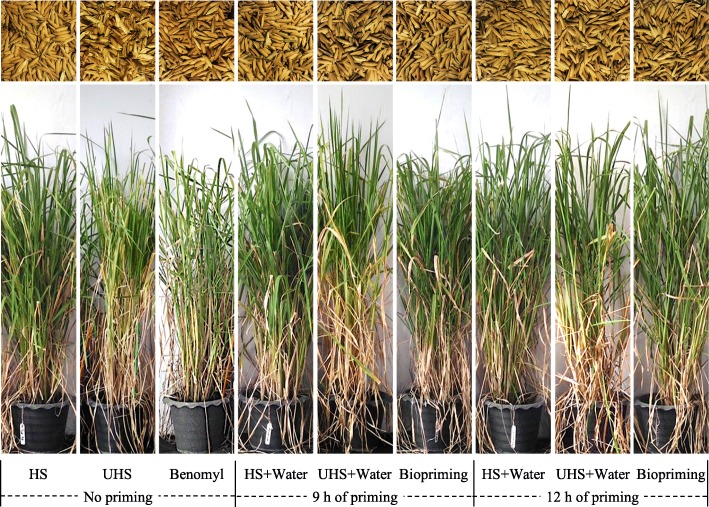
Comparisons of rice plants and yields derived from different conditions tested in pot experiments. Rice plants and yields merging from healthy seeds (HS), unhealthy seeds (UHS), fungicide treated seeds, and primed seeds. The fungicide-treated seeds were prepared using UHS plus chemical fungicide, benomyl (Benomyl). The primed seeds were prepared using HS or UHS primed with water (HS + water or UHS + water) and UHS bioprimed with bacterial cell suspension made of *Bacillus* sp. CZR007 (Biopriming). Seed hydropriming and biopriming were carried out for 9 and 12 h. The pictures of rice plants and their yields were photographed at the harvesting stage in pot experiments

For the pot experiments, due to the mean results derived from unhealthy rice seeds priming with different selected bacteria were not significantly different, we, therefore, pooled all data together and reported as one global mean (Figs. 6 and 7). At every cultivation phase of rice, the height of rice plants increased dramatically from seedling to flowering stages and stopped there (Fig. 6-a). There was no significant difference in the height of rice plants arising from any seeds. Also, the numbers of tillers and panicles per hill of rice plants originating from any seeds were not significantly different (Fig. 6-b and -c). A similar trend of results was observed for the weight of 1000 healthy rice grains produced (Fig. 6-d). However, the percentages of healthy rice yields were not significantly different, when these yields were produced by rice plants emerging from non-treated or hydroprimed healthy seeds and chemical fungicide-treated or bioprimed unhealthy seeds (Figs. 6 and 7-e). Besides, such percentages were lowest for those rice yields produced by rice plants arising from non-treated or hydroprimed unhealthy seeds.

**Fig. 8:**
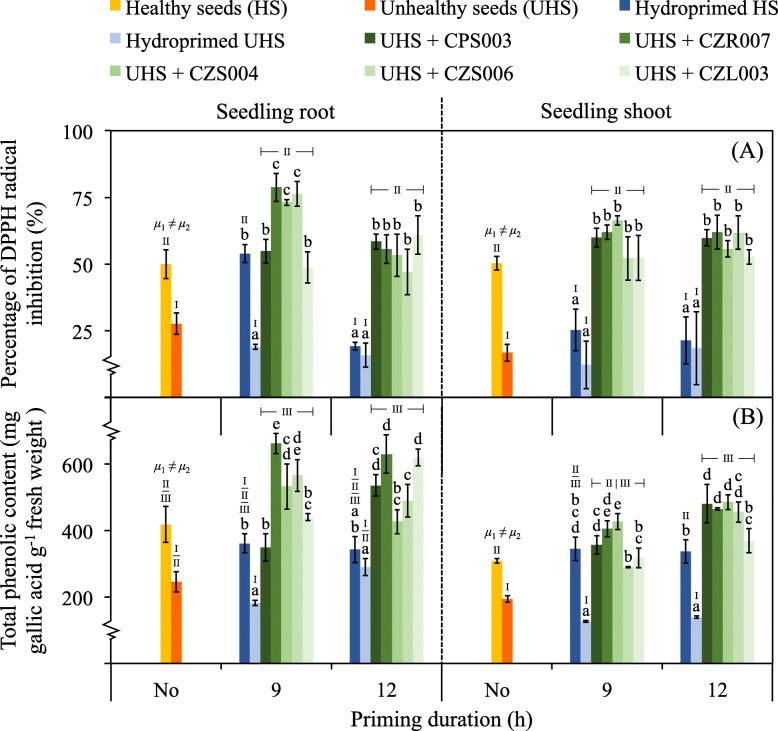
Impacts of seed biopriming on antioxidant and total phenolic contents in rice seedlings. The percentage of DPPH radical inhibition referring to the antioxidant activity (**a**) and the total phenolic content (**b**) were measured in 14-day-old seedlings’ roots and shoots. Five bacterial isolates (CPS003, CZR007, CZS004, CZS006, and CZL003) were used for seed biopriming. Bioprimed unhealthy seeds (UHS) were tested in comparison with the controls made of non-treated (No) or hydroprimed healthy seeds (HS) and UHS. Error bars are mean values ± SDs of at least three replicated measurements. The statistical comparison of means between HS (*μ*1) and UHS (*μ*2) without priming was conducted using the independent-samples *t*-test at *P* = 0.05. The statistical differences of means compared by one-way ANOVA with Tukey’s post hoc test at *P* ≤ 0.05 are indicated with small case letters for every type of primed seeds (within the same priming duration) and with the Roman numbers for the comparison between any tests and controls. The data derived from any bioprimed seeds were calculated as a mean, regardless of the different bacteria tested

**Fig. 9:**
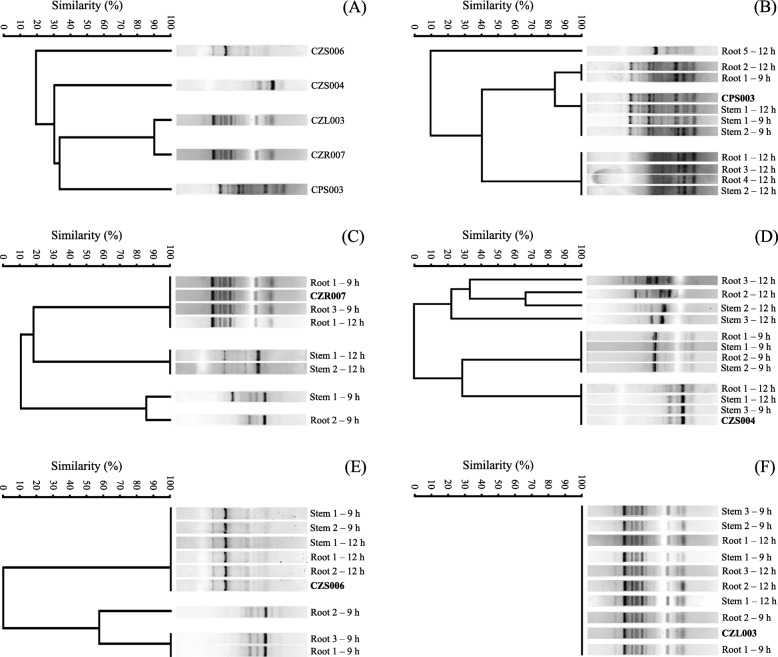
*In planta* colonization of biopriming bacteria. The ERIC-PCR DNA fingerprints obtained from the axenic cultures of any bacterial isolates used for seed biopriming (**a**) are the standards, compared to those other bacteria isolated from rice seedlings arising from bioprimed unhealthy seeds with bacterial isolate CPS003 (**b**), CZR007 (**c**), CZS004 (**d**), CZS006 (**e**), or CZL003 (**f**). The code behind the fingerprint refers to the source (root or shoot) of seedling tissues, following by order of the bacterial colony isolated from such respective source and the priming duration in seed biopriming

The antioxidant activity (referring to the % Diphenyl-1-Picrylhydrazyl (DPPH) radical inhibition) and total phenolic content as the health indicators of rice seedlings are present in Fig. 8. The antioxidant activities in roots of seedlings originating from 12-h hydroprimed healthy seeds and non-treated or any hydroprimed unhealthy seeds were significantly lower than the other conditions (Fig. 8-a). A similar trend of results was observed in the case of seedlings’ shoots, but the antioxidant activity in shoots of seedlings emerging from 9-h hydroprimed healthy seeds decreased. The total phenolic contents in roots and shoots of seedlings were similar to the results of antioxidant activities (Fig. 8-b). The Enterobacterial repetitive intergenic consensus-polymerase chain reaction (ERIC-PCR) fingerprinting data to confirm *in planta* colonization of selected bacteria after seed biopriming are present in Fig. 9. At least a bacterial colony isolated from rice seedlings emerging from bioprimed unhealthy seeds exhibited 100% similarity of its fingerprinting data compared to those of selected bacteria used in seed biopriming.

## Discussion

The paddy field is a huge playground of diverse microorganisms, and these tiny bugs play significant roles that provide either beneficial or adverse impacts on rice crop. When a plant disease occurs in the paddy field, they are easy to be dispersed and hard to be managed. DPD is a common disease of rice seeds that may arise in the productive stage of rice but more often during post-harvest storage. The causative agents of this disease are diverse fungi but are typically the members of the genera *Alternaria*, *Bipolaris*, *Cercospora*, *Curvularia*, *Fusarium*, and *Sarocladium* [2]. Based on our study, the fungus, *Nigrospora* was found for the first time as one among the potential pathogens of DPD. All pathogens damage rice seeds by initiating grain discoloration and consequently destroy the entire grain tissues.

Plant tissues of rice offer unique ecological niches for diverse microbes and some of which are potential PGP bacteria [15–17]. It was unsurprising that our isolated bacterial endophytes of rice, showing excellent antifungal activity against pathogenic fungi of DPD were all the members of the genus *Bacillus*. Many studies report that bacilli are ones among the best-known PGP bacteria of rice [14, 15, 18, 22]. The PGP roles of bacilli in rice cultivation are diverse, which can act as a free-living diazotroph to supply nitrogen to rice, a producer of antimicrobial substances to suppress pest invasion, and a modifier of minerals in rice crop, etc. The different antifungal potentials of our bacilli can be a result of their phylotypic difference as well as the different fungal pathogens tested. It has been reported that variety of bacilli hold different lipopeptide genes which related to the antimicrobial activity, and such produced lipopeptide antibiotics can be those of the surfactin, iturin and fengycin families [23]. Moreover, all isolated bacilli can tolerate several herbicides, which could be an add-on feature to minimize the side effects of herbicide treatment on rice development in the commercial rice cultivation.

After biopriming of unhealthy rice seeds with the selected bacteria, priming duration for 9 or 12 h was optimal, supported by the lowest disease incidence and longer roots and shoots of rice seedlings germinated. The appropriate priming duration is a critical factor in seed priming technique, which affects the germination behavior and seedling vigor of various plant seeds, e.g., pinto bean [24] and bread wheat [25]. Xie et al. [26] found that soaking seeds of rice cultivar 9311 in *Bacillus subtilis* OKB105 for 2 h could increase the length of rice seedlings’ shoots by 25.2%. Another study performing rice seed biopriming with *B. subtilis* CIM and picomolar rutin could enhance also the growth and development of rice seedlings’ roots and shoots [27]. We also found that hydropriming of unhealthy rice seeds could enhance rice development at the germination and seedling growth phases. This finding is concordant with a study unveiling that hydropriming of rice seeds could accelerate emergence and improve rice seedling vigor [28]. However, the results derived from the pot experiments suggested that biopriming of unhealthy rice seeds offered more significant support in the suppression of DPD than those hydropriming ones, evidenced by the better quality of rice yield produced. This finding would be an obvious result of PGP benefits provided by the bacteria used in seed biopriming.

Antioxidant and phenolic compounds are the keystone bioactive phytochemicals of rice, which indicate the nutritive quality of rice and play roles in plant-microbe interactions [17, 29]. Our study revealed that most isolated bacilli used in biopriming of unhealthy rice seeds could statistically increase the two phytochemical contents in rice seedlings regardless of the different priming durations tested. This finding is an example mechanism of how such biopriming bacilli could immunize rice plants. Singh et al. [27] found that biopriming of rice seeds with *B. subtilis* CIM not only enhanced seedling growth but also induced the accumulations of photosynthetic pigments (i.e., chlorophyll and carotenoid) and phenolic and flavonoid contents in rice seedlings. Another study reported that antagonistic *Bacillus* spp. significantly enhanced the activities of antioxidative enzymes (e.g., peroxidase, polyphenol oxidase, phenylalanine ammonia-lyase) up to 4-fold, in leaves and roots of rice growing under hydroponic and soil conditions [30]. Besides, such antioxidative substances have been supposed to be elicitors of the induced systemic resistance in rice as their functions highly correlate with rice disease suppression [31, 32].

Effectively *in planta* colonization of beneficial microbes is a prime reason to prolong their bioactive functions in supporting growth and development of their host plants [13]. The mechanisms by which the endophytic bacteria enter the interiors of rice are similar to those by phytopathogenic bacteria, and some of entry paths like germinating radicles and root cracks assist the initiation of root colonization by bacteria [13]. It was found also that during germination of rice seeds bioprimed with *B. subtilis* OKB105, more than 150 genes of this bacterium exhibited significantly altered transcriptional levels [26]. For example, genes involved in metabolisms, nutrient transport and stress responses (e.g., *araA*, *ywkA*, *yfls*, *mtlA*, *ydgG*, etc.) were upregulated, while *cheV*, *fliL*, *spmA* and *tua* genes involved respectively in chemotaxis, motility, sporulation and teichuronic acid biosynthesis were downregulated [26]. The ERIC-PCR DNA fingerprinting technique carried out in our study proved that all isolated bacilli used in seed biopriming could extend their lives in the interiors of rice seedlings. Hence, the improved rice yields produced in the pot experiments by using seed stocks made of unhealthy rice seeds bioprimed with herbicide-tolerant endophytic bacteria would be a result of prolonged synergistic interactions between rice plants and the biopriming bacteria.

## Conclusions

Biopriming of unhealthy rice seeds with herbicide-tolerant endophytic bacteria of rice could suppress DPD of rice seeds. The synergistic interactions between biopriming bacteria and rice seeds optimized the growth and development of rice generated in subsequent cultivation, leading to the acceptable quality of rice yield produced. Therefore, seed biopriming would be a promising approach in the restoration of unhealthy rice seeds for use as rice seed stock, which underpins the use of green technology in sustainable agriculture of rice.

## Methods

### Plant materials

Any plant materials used in this study were identified by a curator, Tanawat Chaowasku and deposited publicly as herbariums for further references at the CMUB Herbarium, Department of Biology, Faculty of Science, Chiang Mai University, while their specimen voucher numbers initiated with CMUB are addressed elsewhere in this article.

Thai jasmine rice (*O. sativa* L. cv. KDML105) seeds (CMUB39907) stored without any treatment for 4–5 months after harvesting in a granary at Si Prachan District, Suphan Buri Province, Thailand. These seeds were commercially produced by a local farmer, and some of which were stored as seed stock for subsequent cultivation. The seeds were collected from the granary with the owner’s permission and guidelines (no license needed), and they showed either apparent or no symptom of DPD. We classified the virulence degrees of DPD of these seeds into Grade A (healthy): no disease symptom, Grade B (unhealthy): 20–30% visible symptom, and Grade C (diseased): 50–100% severe seed damage. The seeds were the sources for isolation of phytopathogenic fungi and used in biopriming experiments.

Two rice cultivars: *Oryza sativa* L. var. *indica* cv. Pathumthani 1 (CMUB39903 – CMUB39905) and *Oryza sativa* L. var. *indica* cv. RD41 (CMUB39906) were commercially cultivated by the local farmers at different field locations where different herbicides were applied to treat the fields (Table 2). These field-growing rice plants were collected by uprooting from the fields with the owners’ permission and guidelines (no license needed). The farmers also kindly provided the information about the herbicide treatments and the ages of rice plants. The rice plants were packed in plastic bags and preserved in an icebox before transferring to the laboratory. After washing these rice plants several times under running tap water, they were divided as roots, stems, and leaves. Any plant materials had surfaces sterilized by soaking in a series of 70% (v/v) ethyl alcohol for 1 min, 2% (v/v) NaOCl for 2 min, 95% (v/v) ethyl alcohol for 30 s, and 30% (v/v) H_2_O_2_ for 1 min. The cleaned plant materials were washed four times with sterile distilled water to remove disinfectant residues and used as the sources for isolating herbicide-tolerant endophytic bacteria.

### Isolation of phytopathogenic fungi and their pathogenicity tests

Diseased rice seeds (Grade C) served as the sources for isolation of fungal pathogens causing DPD. These seeds were washed several times under running tap water and their surfaces sterilized with 1% (w/v) NaOCl for 10 min following by a final wash with sterile distilled water. Ten cleaned seeds were pasted on potato dextrose agar (PDA) medium (Difco, USA) and incubated at 22 °C for 5–8 days. The appeared fungal colonies were subcultured on the new PDA medium until becoming axenic cultures and then preserved in 20% (v/v) glycerol for storage and further studies.

Pathogenicity of isolated fungi was tested in vitro at germination and seedling growth phases of healthy rice seeds (Grade A), following Koch’s postulates. Briefly, these seeds had their surfaces sterilized using the same protocol addressed before. For the germination test, five of the cleaned seeds were pasted on autoclaved planting paper and inoculated with 10 mL of sterile distilled water as the control or mycelial suspension made of each fungal isolate, then incubated at 30 °C. The mycelial suspension was prepared by scrapping 7-day-old fungal biomass growing on PDA medium at 30 °C. The biomass was ground in the presence of sterile distilled water, using aseptic mortar and pestle. The final concentration of such mycelial suspension was adjusted to 10^4^ propagules mL^− 1^ by hemocytometer.

For the test at the seedling growth phase, the cleaned rice seeds were allowed to germinate on moist autoclaved planting papers at 30 °C for 5 days. A seedling produced was aseptically transferred to grow further on Water agar medium in a test tube, where 1 mL of sterile distilled water (control) or each mycelial suspension was inoculated and incubated in a moist chamber at 25 °C. The pathogenicity level (i.e., weak, medium, and virulent) of each isolated fungus was assigned using the germination rate of seeds and seedling health observed every day.

### Isolation of herbicide-tolerant endophytic bacteria of rice and their antifungal potentials

The prepared rice plant materials derived from the field cultivations were ground in the presence of sterile distilled water (1 mL), using aseptic mortar and pestle. The suspension (100 μL) of ground plant materials was spread over Luria-Bertani (LB) agar medium (Himedia, India) supplemented with a set of herbicides applying in the field cultivation of rice (Table 2). Also, the washing liquid derived from the last step of the surface sterilization of each plant material described previously (100 μL) was spread over the agar medium and served as a control to verify *in planta* origin of the bacteria isolated. Four seeded agar plates per each part of plant materials and controls were carried out and incubated at 30 °C for 2 days. The visible bacterial colonies were subcultured onto the new agar medium until becoming axenic cultures, then preserved in 20% (v/v) glycerol for storage and further studies.

Antifungal activity of each isolated bacterium against a set of fungal isolates showing virulent pathogenicity of DPD was assayed using dual culture method on PDA medium. Briefly, a bacterial colony (2-day-old culture growing previously on LB agar medium at 30 °C) was streaked (5-cm in length) on PDA medium at 2-cm away from the edge of the plate. At the opposite of the bacterial streak, the fungal mycelium as a disc of 5.5 mm diameter (5-day-old culture growing previously on PDA medium at 30 °C) was inoculated at 2-cm away from the edge of the plate. All assayed plates were incubated at 30 °C, in which the radius growth of fungal colony was measured every day. Fungal growth in the absence of test bacterium served as the control. The percentage of inhibition was calculated using equation (i), where *d*_control_ is an average size (ø in mm) of the fungal colony in the control and *d*_assay_ is that in the dual culture assay. $\%\mathrm{Inhibition}=\frac{d_{\mathrm{control}}-{d}_{\mathrm{assay}}}{d_{\mathrm{control}}}\times 100$ (i).

### Identification and classification of isolated microbes

The isolated fungi showing virulent pathogenicity of DPD were identified at the generic level, using their internal transcribed spacer (ITS) gene sequence data. Each fungus was grown on PDA medium at 30 °C for 7 days, in which the fungal biomass was scraped out and suspended in sterile distilled water. The biomass collected by centrifugation at 12100 *g* for 5 min was used for DNA extraction with Plant Genomic DNA Mini Kit (Geneaid Biotech Ltd., Taiwan), following the manufacturer’s instruction. Extracted DNA was the template for amplifying the fungal ITS1–5.8S rDNA – ITS2-26S rDNA region by PCR, using *Taq* DNA Polymerase and Standard *Taq* Buffer from New England BioLabs, USA. PCR (25 μL) contained 2.5 μL of 10× Standard *Taq* Reaction Buffer, 0.5 μL of 10 mM dNTPs, 0.125 μL of *Taq* DNA Polymerase, 0.5 μL of each 10 μM primer (ITS1: 5′ TTTCCGTAGGTGAACCTGC 3′ and ITS4: 5′ TCCTCCGCTTATTGATATGC 3′ [33]), 0.1 μL of template DNA, and the volume was adjusted with nuclease-free water. PCR was carried out using a thermocycler with the following condition: 5 min initial denaturation at 94 °C, 30 cycles of 1.5 min denaturation at 94 °C, 2 min annealing at 52 °C and 1 min extension at 72 °C, and 5 min final extension at 72 °C.

The isolated bacteria showing excellent antifungal activity were identified at the generic level, using their 16S rRNA gene sequence data. Each bacterium was grown in 50 mL of LB broth with shaking incubation at 250 rpm, 30 °C for 2 days. The bacterial biomass was collected by centrifugation at 12000 *g* for 5 min and used as the template DNA for amplifying the 16S rRNA gene sequence. Following the same PCR compositions mentioned above, except for the primers that were fD1 5′ AGAGTTTGATCCTGGCTCAG 3′ and rP2 5′ ACGGCTACCTTGTTACGACTT 3′ [34]. PCR was carried out using a thermocycler with the following condition: 5 min pre-denaturation at 95 °C, 30 cycles of 1 min denaturation at 94 °C, 1 min annealing at 55 °C and 1.5 min extension at 72 °C, and 10 min final extension at 72 °C.

Any PCR products were sequenced to retrieve their nucleotide sequence data with a service provided by 1st BASE, Singapore. The gene sequences obtained were checked using BioEdit (www.mbio.ncsu.edu/BioEdit/bioedit.html) and identified by comparison with the public nucleotide databases available in GenBank (https://blast.ncbi.nlm.nih.gov/Blast.cgi) plus MycoBank (http://www.mycobank.org) for the fungal ITS sequences or together with EZBioCloud (www.ezbiocloud.net) for the bacterial 16S rRNA gene sequences. The highly related nucleotide sequences were collected, aligned with MUSCLE, and used for constructing phylogenetic trees in MEGA7 (www.megasoftware.net).

### Seed biopriming

The method for biopriming of rice seeds was carried out using modified protocols described by Singh et al. [27] and Sivakumar et al. [35]. Briefly, unhealthy rice seeds (Grade B) were used for biopriming by soaking them in aqueous suspensions of the isolated bacteria showing excellent antifungal activity. Each bacterium was previously grown in LB broth at 30 °C with shaking at 250 rpm for 2 days, and its biomass was collected by centrifugation at 9660 *g* for 5 min. The bacterial biomass was washed twice, re-suspended and adjusted its optical density (absorbance) at the wavelength of 600 nm using sterile distilled water to 0.2 that corresponded to 10^8^ colonies forming unit per mL. The seeds were soaked in each bacterial suspension for 3, 6, 9, 12, and 15 h, aiming to find the OPD in biopriming. All soaked seeds were air dried at 25 °C till their moisture contents were the same as before priming (~ 12%) monitored by a rice moisture meter, RICETER F-514 (Kett Electric Laboratory, Japan) to avoid the impacts of seed moisture change caused by different priming durations. In parallel, a set of controls was constructed to assess the capacity of seed biopriming, i.e., non-treated or hydroprimed healthy and unhealthy seeds. For seed hydropriming, it was carried out in the same way as for biopriming but used solely sterile distilled water (no bacterial cells). Besides, unhealthy seeds mixed with 1.3% (w/w) Benomyl (Sims Agrow Cheme, Thailand) served as a chemical fungicide-treated control in pot experiments. Any rice seeds with and without treatment were kept at 4 °C for 6 months before use in further assessments.

### Post-biopriming assessments in the full life cycle of the rice crop

The impacts of seed biopriming on fertility recovery and disease suppression of unhealthy rice seeds, since seed germination until harvesting of rice yield were evaluated. GP, GI, MGT, DI, and lengths of seedlings’ roots and shoots, were evaluating factors at the germination and seedling growth phases and also used as the criteria for assigning OPD in biopriming. These parameters were quantified after allowing seeds to germinate by a standard between-paper germination test described in the International Seed Testing Association’s handbook [36]. Non-treated, hydroprimed or bioprimed healthy and unhealthy rice seeds previously prepared were used in these measurements, which were carried out in four replications (100 seeds per each) and incubated at 25 °C in plastic bags to prevent humidity loss.

The germinated seeds counting for the first time on the third day of incubation and the seventh day for the last count were recorded and subjected to the calculation of GP [36]. GI was calculated using the equation (ii) described in the Association of Official Seed Analysts’ handbook [37], in which *G*_*x*_ is the number of germinated seeds counting at day *x*.

ii $$ \mathrm{GI}=\frac{G_1}{1}+\frac{G_2}{2}+\dots +\frac{G_x}{\mathrm{x}} $$

The equation (iii) described by Ellis and Roberts [38] was used to calculate MGT, where *n* is the number of seeds germinating on day *d* and *d* is the number of days counting from the beginning of germination.

iii $$ \mathrm{MGT}=\frac{\sum d\cdot n}{\sum n} $$

At the seventh day after allowing rice seeds to germinate, DI scores were determined using the grain discoloration criteria of Mew and Misra [39] as no incidence = 0, < 1% = 1, 1–5% = 3, 6–25% = 5, 26–50% = 7, and 51–100% = 9. Based on this scoring, DI was computed using the equation (iv), where each *N*_3_, *N*_5_, *N*_7_, and *N*_9_ is respectively the number of seedlings with score 3, 5, 7, and 9, and *N*_*t*_ is the total number of scored seedlings.

iv $$ \mathrm{DI}=\frac{N_3+{N}_5+{N}_7+{N}_9}{N_t} $$

Lengths of seedlings’ roots and shoots were measured using 14-day-old rice seedlings emerging from any treated and control seeds.

The pot experiments were constructed to assess how seed biopriming benefits rice cultivation and quality of rice yield produced. The tests were conducted with a randomized complete block design with five replications (4 pots per each) under the controllable greenhouse conditions. Each container (ø = 8 in, height = 25 cm) was filled with 2 kg of agricultural soil homogenized with coir and cow manure at a ratio of 4/1/1 (w/w/w). The soil type was clayey loam identified by the hydrometer method [40]. The 14-day-old rice seedlings emerging from either non-treated or treated seeds were prepared in the same way as addressed previously. At least three seedlings were planted at the center of each pot and allowed to grow for 5 days when only the best-grown seedling was remained. With the aim to imitate the field conditions for commercial rice cultivation, every pot was fertilized twice with 30 g of Nitrogen-Phosphorus-Potassium (N46-P0-K0) fertilizer for the first time (20 days after planting) and 60 g of N16-P16-K16 for the last time (45 days after planting). The field concentration of each fertilizer denoted by farmers was 30 and 60 kg ha^− 1^ for N46-P0-K0 and N16-P16-K16, respectively. A set of herbicides was also applied twice by spraying on soil surrounding rice plants when they were 10 and 25 days old. The herbicides used were 0.64 mL L^− 1^ Clomazone plus 1.44 mL L^− 1^ Propanil for the first time and 0.276 mL L^− 1^ Fenoexprop-P-Ethyl for the second time. The growth phases of rice after planting comprised of seedling (day 0–14), tillering (day 30–60), flowering (day 75–100), and harvesting (day 120). Growth and health indexes of rice, including the height of rice plants at every growth phase and the numbers of tillers and panicles per hill, the weight of 1000 healthy rice grains and the percentage of healthy rice yield at the harvesting stage were measured and used to determine the benefits of seed biopriming.

### Analyses of seedling phytochemicals

As well-being indicators, two phytochemicals, i.e., antioxidants and total phenolic contents in rice seedlings’ roots and shoots were quantified using modified colorimetric methods described by Sadh et al. [41]. Non-treated, hydroprimed or bioprimed healthy and unhealthy rice seeds previously prepared were used to produce 14-day-old seedlings. A modified DPPH radical scavenging assay was used to measure antioxidant activity. Briefly, 500 mg of seedlings’ shoots or roots was extracted using 2 mL of 95% (v/v) ethanol, and the mixture was precipitated by cold centrifugation (4 °C) at 9660 *g* for 20 min. The derived supernatant or the extracting solvent as a control (0.5 mL) was mixed with 3 mL of 60 M DPPH by vortexing and incubated in the dark at 25 °C for 30 min. The absorbance at 517 nm (A_517_) of the produced mixture was measured by a spectrophotometer, while the antioxidant activity was determined with the percentage of DPPH radical inhibition, using the modified equation (v).

v $$ \%\mathrm{DPPH}\ \mathrm{radical}\ \mathrm{inhibition}=\frac{A_{517\ \mathrm{control}}-{A}_{517\ \mathrm{extract}}}{A_{517\ \mathrm{control}}}\times 100 $$

For the quantification of the total phenolic contents, 50 mg of seedlings’ shoots or roots was extracted by mixing with 2.5 mL of 95% (v/v) ethanol and incubated at 0 °C for 48–72 h. The extract was combined using a homogenizer and precipitated by cold centrifugation (4 °C) at 9660 *g* for 10 min. The derived supernatant or the extracting solvent as a control (1 mL) was mixed with 2.5 mL of 95% (v/v) ethanol, 5 mL of sterile distilled water, and 0.5 mL of 50% (v/v) Folin-Ciocalteu reagent, by vortexing and left at 25 °C for 5 min. The mixture was added with 1 mL of 5% (v/v) Na_2_CO_3_ and incubated in the dark at 25 °C for 1 h. The absorbance at 725 nm of the produced mixture was measured by a spectrophotometer, while the total phenolic content in mg gallic acid per g fresh weight of plant materials was calculated by corresponding the absorbance values to the standard curve of the known gallic acid concentrations.

### Confirmation of *in planta* colonization by herbicide-tolerant endophytic bacteria

The capability of PGP bacteria to colonize the interiors of rice is a promising feature that these bacteria can benefit the entire life cycle of rice growth and development. ERIC-PCR [42] was carried out to confirm the *in planta* colonization of the bacteria selected for seed biopriming. Briefly, surface-sterilized roots and shoots of 14-day-old rice seedlings emerging from bioprimed seeds were prepared as described previously. The cleaned plant materials (1 g) were ground in the presence of sterile distilled water (9 mL), using aseptic mortar and pestle. The ground plant suspension (1 mL) was diluted 10-fold serially, and 100 μL of some dilutions (10^3^–10^5^) was spread over LB agar medium supplemented with a set of herbicides used for the bacterial isolation. The experiments were carried out in triplicate, and the growing bacterial colonies were collected randomly and used as a template DNA for ERIC-PCR, while axenic cultures of the bacteria selected for seed biopriming served as the controls. A pair of primers [43], including ERICIR (5′-ATGTAAGCTCCTGGGGATTCAC-3′) and ERIC2 (5′-AAGTAAGTGACTGGGGTGAGCG-3′) was used in ERIC-PCR, in which the other PCR compositions were the same as mentioned before. ERIC-PCR was performed using a thermocycler with the following condition: 5 min pre-denaturation at 95 °C, 30 cycles of 1 min denaturation at 94 °C, 1 min annealing at 52 °C and 1 min extension at 72 °C, and 10 min final extension at 72 °C. The PCR product (10 μL) was analyzed using 1.5% (w/v) agarose gel electrophoresis at 100 V for 3 h. The DNA fingerprint on the gel was viewed and imaged by a Gel Doc™ XR+ Gel Documentation System (Biorad, USA), after staining with 0.5 g mL^− 1^ ethidium bromide. The fingerprints derived from different bacterial colonies and controls were clustered and analyzed for their similarity percentage by using PAST version 3.20 (https://folk.uio.no/ohammer/past/) [44].

### Statistical analysis

Comparisons of mean values and standard deviations (SDs) obtained from any measurements were performed using the independent-samples *t*-test or analysis of variance (ANOVA) with Tukey’s post hoc tests, all available in the SPSS version 25.0 (SPSS, Chicago IL, USA) software package. The statistical results and significance (*P*) levels (*P* ≤ 0.05) are addressed elsewhere in this article.

## Data Availability

All data generated during this study are included in this published article. The nucleotide sequences derived from the isolated microbes in this study are publicly available at GenBank (https://www.ncbi.nlm.nih.gov/genbank/) with accession numbers; MG309751-MG309756 for fungal isolates (Table 1 and Fig. 1) and MG309712-MG309716 for bacterial isolates (Table 3 and Fig. 3).

## Abbreviations

ANOVA: Analysis of variance
DI: Disease incidence
DNA: Deoxyribonucleic acid
DPD: Dirty panicle disease
DPPH: Diphenyl-1-Picrylhydrazyl
ERIC-PCR: Enterobacterial repetitive intergenic consensus-PCR
GI: Germination index
GP: Germination percentage
ITS: Internal transcribed spacer
LB: Luria-Bertani
MGT: Mean germination time
OPD: Optimal priming duration
PCR: Polymerase chain reaction
PDA: Potato dextrose agar
PGP: Plant growth-promoting
